# Increased mortality and altered local immune response in secondary peritonitis after previous visceral operations in mice

**DOI:** 10.1038/s41598-021-95592-5

**Published:** 2021-08-10

**Authors:** Jonas Menz, Laura Hundt, Tobias Schulze, Katrin Schmoeckel, Pia Menges, Grazyna Domanska

**Affiliations:** 1 Department of General, Visceral, Thoracic and Vascular Surgery, University Medicine Greifswald, Ferdinand-Sauerbruch-Straße, 17475 Greifswald, Germany; 2 Immunology Department, Institute of Immunology and Transfusion Medicine, University Medicine Greifswald, Greifswald, Germany

**Keywords:** Immunology, Pathogenesis, Experimental models of disease

## Abstract

Postoperative peritonitis is characterized by a more severe clinical course than other forms of secondary peritonitis. The pathophysiological mechanisms behind this phenomenon are incompletely understood. This study used an innovative model to investigate these mechanisms, combining the models of murine *Colon Ascendens Stent Peritonitis* (CASP) and *Surgically induced Immune Dysfunction* (SID). Moreover, the influence of the previously described anti-inflammatory reflex transmitted by the vagal nerve was characterized. SID alone, or 3 days before CASP were performed in female C57BL/6 N mice. Subdiaphragmatic vagotomy was performed six days before SID with following CASP. The immune status was assessed by FACS analysis and measurement of cytokines. Local intestinal inflammatory changes were characterized by immunohistochemistry. Mortality was increased in CASP animals previously subjected to SID. Subclinical *bacteremia* occurred after SID, and an immunosuppressive milieu occurred secondary to SID just before the induction of CASP. Previous SID modified the pattern of intestinal inflammation induced by CASP. Subdiaphragmatic vagotomy had no influence on sepsis mortality in our model of postoperative peritonitis. Our results indicate a surgery-induced inflammation of the small intestine and the peritoneal cavity with bacterial translocation, which led to immune dysfunction and consequently to a more severe peritonitis.

## Introduction

Visceral surgery is associated with a high risk of infectious complications, such as postoperative peritonitis (POP)^1–3^. POP frequently develops into abdominal sepsis, a condition with mortality rates of up to 50%. Despite improvements in outcome, sepsis still accounts for about 11 million deaths worldwide each year^4^.

Postoperative peritonitis is a subtype of secondary peritonitis^5^. In general, secondary peritonitis is the result of compromised integrity of the intestinal wall. This injury can be either a result of spontaneous perforation secondary to local inflammation or tumor growth or secondary to iatrogenic manipulation. The latter scenario is the case in postoperative peritonitis, where the damage to the intestinal wall is surgery-related, e.g., following direct perforation or anastomotic leakage (AL). Compared to spontaneous secondary peritonitis, the postoperative subtype shows greater mortality^6^. Increasing rates of comorbidities are proven to be among the risk factors for severe postoperative complications^6–8^. Additionally the impaired immune responses following surgery seems to contribute to the more severe courses of POP^9,10^. The postoperative immunosuppression is characterized by reduced expression of human leucocyte antigen on monocytes, dysfunctional T-cells and a suppressed pro-inflammatory cytokine response ex vivo^6,11,12^. The mechanisms by which the suppressed immune system translates into higher sepsis mortality are still incompletely understood.

In two out of three cases AL is the cause of POP^10,13^. AL in the gastrointestinal tract can occur any time after surgery, from the first to the 40th postoperative day and beyond^7,8^. AL can be divided into early and late AL, depending on the time interval between surgery and diagnosis. Growing evidence suggests that an earlier cut-off point might be more accurate for the definition of early and late AL, based on their underlying mechanisms^8^. Spärreboom et al*.* defined early AL as occurring before the sixth postoperative day. Most frequently, it is due to technical failures and associated with complex procedures or medical emergencies^8^. In contrast, late AL results from delayed wound healing secondary to suboptimal perfusion of the anastomosis or the presence of risk factors for impaired wound healing (for example, preoperative radiotherapy)^14^. Thus, the risk factors for late AL are more frequently patient-related and less predictable^7,8^. Since most studies found AL to occur between the 6th and 12th postoperative day, late AL might be the more common form following this definition^15–19^.

Most murine models for secondary peritonitis directly induce stool leakage into the peritoneal cavity^20–26^. However, this does not reflect the clinical reality of AL since, in this condition, the infectious insult usually impacts the peritoneal cavity only a few days after an initial surgical trauma that induced profound changes of the local immune homeostasis^27–29^. To date, the immune conditions in POP are not as well investigated as those of other forms of secondary peritonitis. In order to obtain a murine model that better reflects the modifications of the peritoneal immune homeostasis after initial surgery we combined two murine models: *Surgically induced Immune Dysfunction* (SID)^12^ and *Colon Ascendens Stent Peritonitis* (CASP)^30^ performed three days later.

Current findings suggest a protective effect of the vagal nerve in murine models of abdominal sepsis^31–34^. Additionally Bonaz et al*.* reviewed the current implications of vagal stimulation in clinical situations. The group proposed beneficial effects of vagal stimulation in situations where high levels of tumor necrosis factor alpha (TNF α) promote clinical symptoms, e.g. inflammatory bowel diseases, rheumatoid arthritis and abdominal sepsis^35^. Via neuroimmunological pathways, acetylcholine released from vagal nerve fibers activates cholinergic receptors on immune cells of the gastrointestinal tract and in subtypes of splenic macrophages^36,37^. Among other effects, this cholinergic activation suppresses TNF α production by these cells and protects the organism from overwhelming inflammation, thus reducing clinical signs of sepsis or chronic inflammation^35^. Furthermore, the proinflammatory response of local immune cells inside the gastrointestinal wall is controlled by vagus nerve stimulation. The latter leads to lower levels of local nitric oxide and prostaglandins, resulting in amelioration of postoperative ileus formation^38^. In line with those findings, loss of the vagal nerve leads to increased mortality with high serum TNF α in rodent models of abdominal sepsis and endotoxemia^31–34^. These studies indicate that the vagal nerve might be a potential target to influence the outcome of POP.

The present study aimed to investigate the effect of a recent surgical trauma on the outcome of peritonitis due to AL and further assessed an impact of subdiaphragmatic vagotomy (VGX) on the outcome of postoperative peritonitis.

## Results

### Prior surgical trauma but not subdiaphragmatic vagotomy lead to higher mortality in secondary murine polymicrobial peritonitis

**Figure 1:**
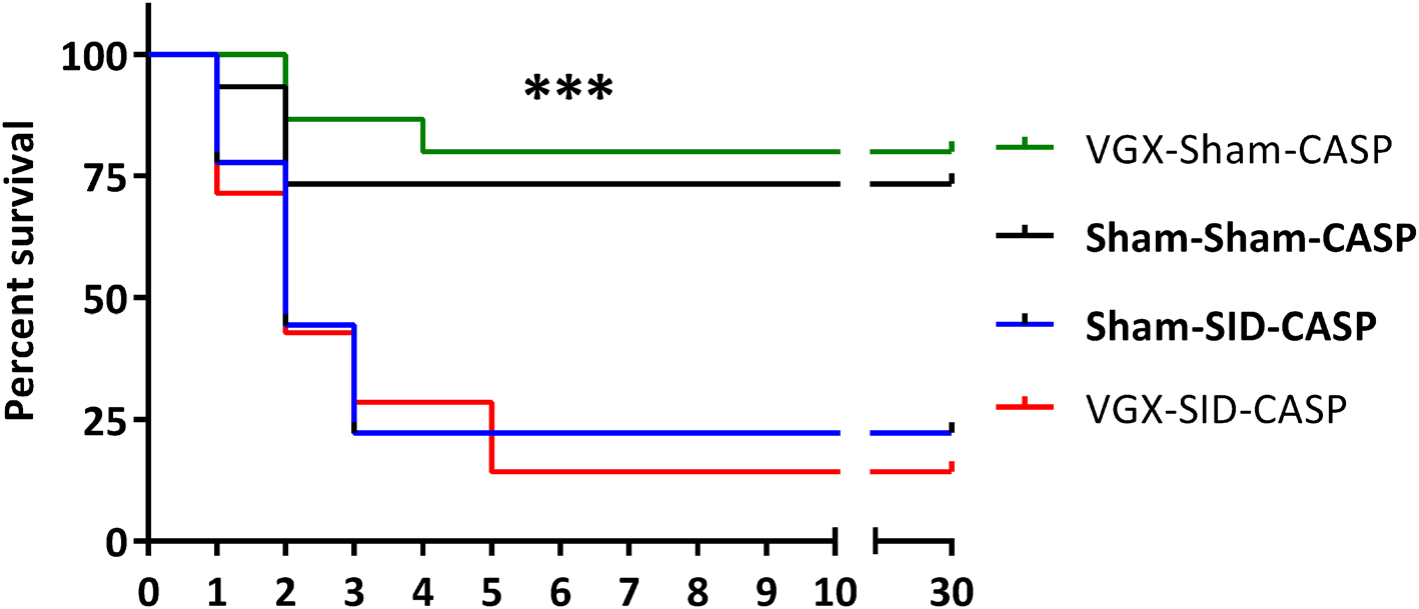
Survival analysis of 18G CASP after vagotomy and/or *Surgically induced Immune Dysfunction.* C57BL/6 N mice underwent subdiaphragmatic vagotomy (VGX) or laparotomy (Sham). After a recovery period of 6 days, either *Surgically induced Immune Dysfunction* or another laparotomy (sham) was performed. Three days later, 18G CASP was performed and mice were controlled every 6 to 8 h the first 2 days and twice per day for 30 days total. The Log-rank Test was performed to determine survival differences between all groups. ***p < 0.001; n = 7 to 15 (mice that have died before or during CASP procedure, as well as technical failures have been excluded).

We have previously shown increased sepsis mortality after vagotomy, suggesting a protective effect of vagal nerve activity during sepsis^32^. In the following experiments, the effect of subdiaphragmatic vagotomy (VGX) on the outcome of postoperative peritonitis in a combined murine model was analyzed. While all mice that have undergone SID showed significantly increased mortality rates, there were no statistically significant differences between animals with and without VGX (Fig. 1 and Supplemental Table 1). Since previous vagotomy had no impact on survival in secondary peritonitis in our disease model, the following studies were performed in animals without prior VGX.

### Surgical trauma leads to reduced peritoneal immune cell counts after 72 h

**Figure 2:**
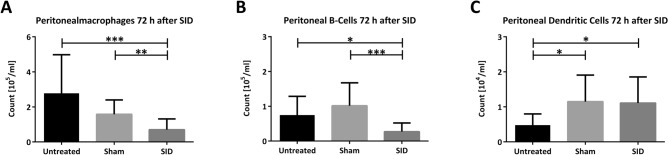
Flow Cytometry analysis of immune cells in peritoneal lavage. C57BL/6 N mice underwent SID operation or laparotomy (sham), and after 72 h peritoneal lavage was harvested and stained for (**A**) macrophages (CD11b^+^ Ly6G^-^), (**B**) B-cells (CD19^+^) and (**C**) dendritic cells (CD11b^+^, CD11c^+^). N = 20 to 22 (technical failures have been excluded); Dunn’s multiple comparison test after significance in Kruskal–Wallis test; *p < 0.05; **p < 0.01; ***p < 0.001.

To characterize the impact of SID on the peritoneal immune homeostasis at the moment of CASP induction, the following analyses were performed 72 h after SID. At this time point, significantly reduced peritoneal macrophage (CD11b^+^ Ly6G^-^) and CD19^+^ B-cell counts were present in the SID group compared to sham-operated mice (Fig. 2). Dendritic cell (CD11b^+^ CD11c^+^) numbers were significantly higher in the peritoneal lavage of both sham and SID groups than in untreated mice. There was no difference in DC count between sham and SID treated mice.

### Surgical trauma leads to bacterial translocation and systemic spread

**Figure 3:**
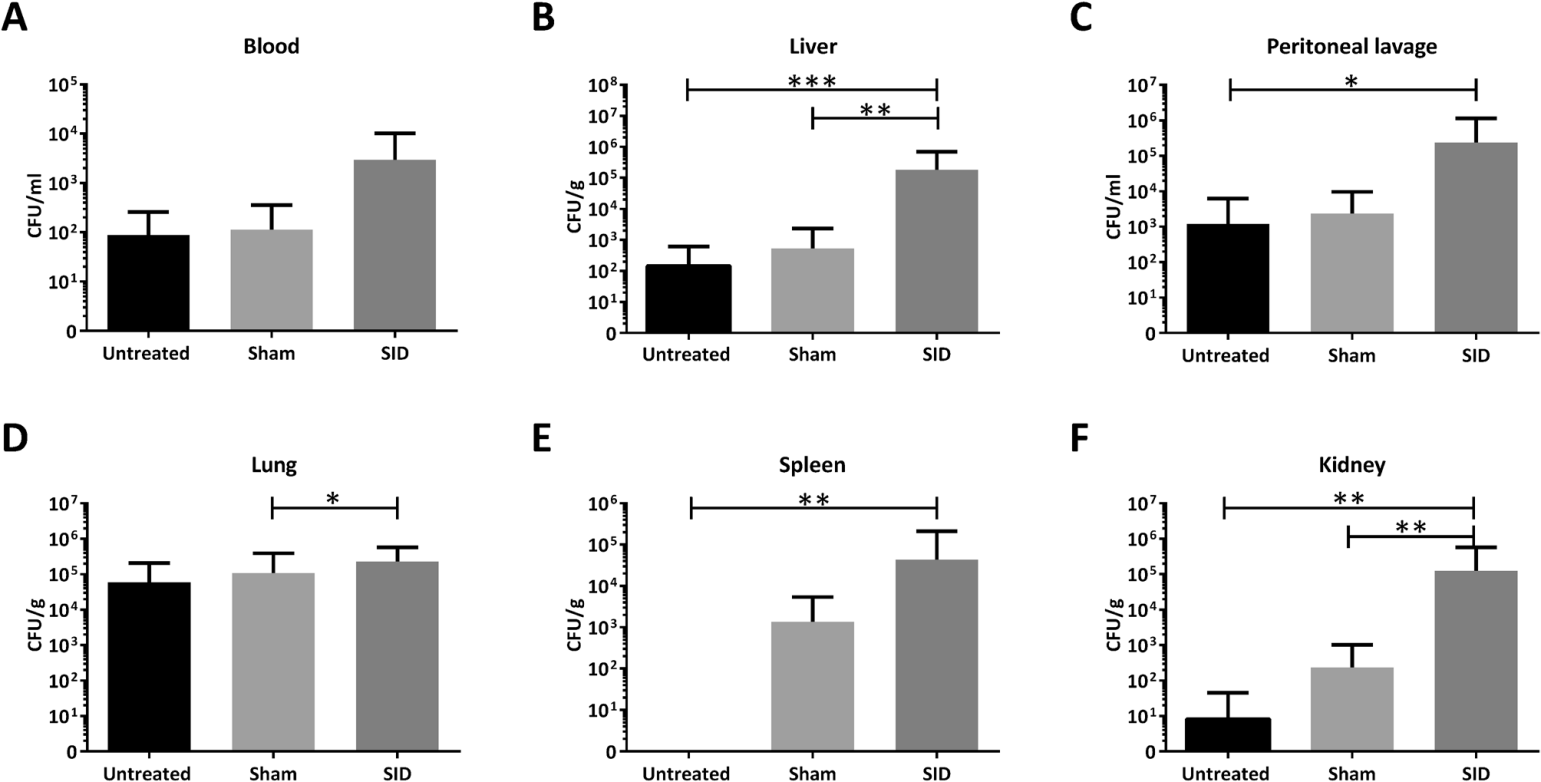
Bacteriological analysis 72 h after *Surgically induced Immune Dysfunction* of peritoneal lavage, blood, liver, lung, spleen and kidney. C57BL/6 N mice underwent SID or the sham operation. After 72 h, colony forming units of (**A**) blood, (**B**) liver, (**C**) peritoneal lavage, (**D**) lung, (**E**) spleen and (**F**) kidney were determined. n = 22 to 24; Mann–Whitney U-test; *p < 0.05; **p < 0.01; ***p < 0.001.

Next, we examined whether surgical manipulation of the gastrointestinal tract led to bacterial translocation and dissemination. Seventy-two hours after SID or sham treatment significantly higher CFU counts were found in the lung, liver and kidney of the SID group compared to sham-treated animals. No significant differences were seen between the bacterial count of untreated and sham-treated animals (Fig. 3).

### SID followed by CASP showed lower numbers of peritoneal immune cells and high IL 10 levels after 24 h

**Figure 4:**
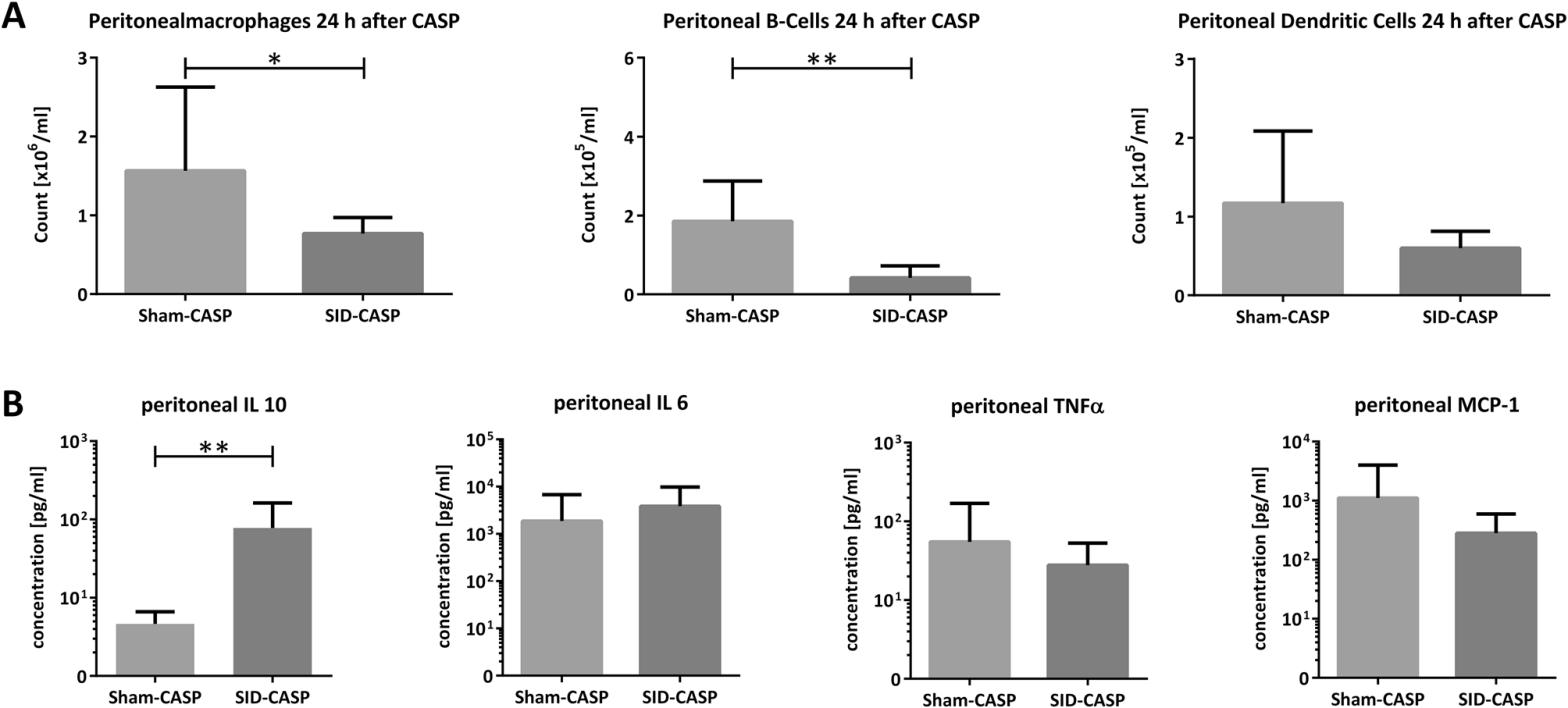
Peritoneal immune status 24 h after CASP. Previously SID- or sham-operated mice underwent 18G CASP, and peritoneal lavage was analyzed 24 h later. (**A**) Flow Cytometry after staining for (**A1**) macrophages (F4/80 +), (**A2**) B-cells (CD19 +) and (**A3**) dendritic cells (CD11b + , CD11c +). (**B**) Supernatant of peritoneal lavage was analyzed for cytokines using CBA with beads against (**B1**) IL 10, (**B2**) TNF α, (**B3**) IL 6 and (**B4**) MCP-1. n = 6 to 7 (mice that have died before or during CASP procedure have been excluded); Mann–Whitney U-Test; *p < 0.05; **p < 0.01; ***p < 0.001; ****p < 0.0001.

Twenty-four hours after CASP, significantly lower counts of peritoneal macrophages and B-cells were found in mice that underwent SID followed by CASP compared to sham followed by CASP. Moreover, there was a trend toward lower dendritic cell counts in SID pretreated animals (Fig. 4A).

To investigate the functional status of the peritoneal immune system twenty cytokines and chemokines were quantified in the peritoneal lavage fluid. Twenty-four hours after CASP, IL 10 levels were significantly higher in mice that underwent SID followed by CASP compared to previous sham followed by CASP. In contrast, no significant differences between SID and sham-operated mice were found for IL 6, TNF α, and MCP-1 levels (Fig. 4B). The other measured cytokines (IFNγ, IL 1ß, IL 4 and IL 17A) were under the detection limit. Untreated control showed no detectable peritoneal cytokine levels in general.

### SID followed by CASP leads to severe inflammation in the small intestinal wall

**Figure 5:**
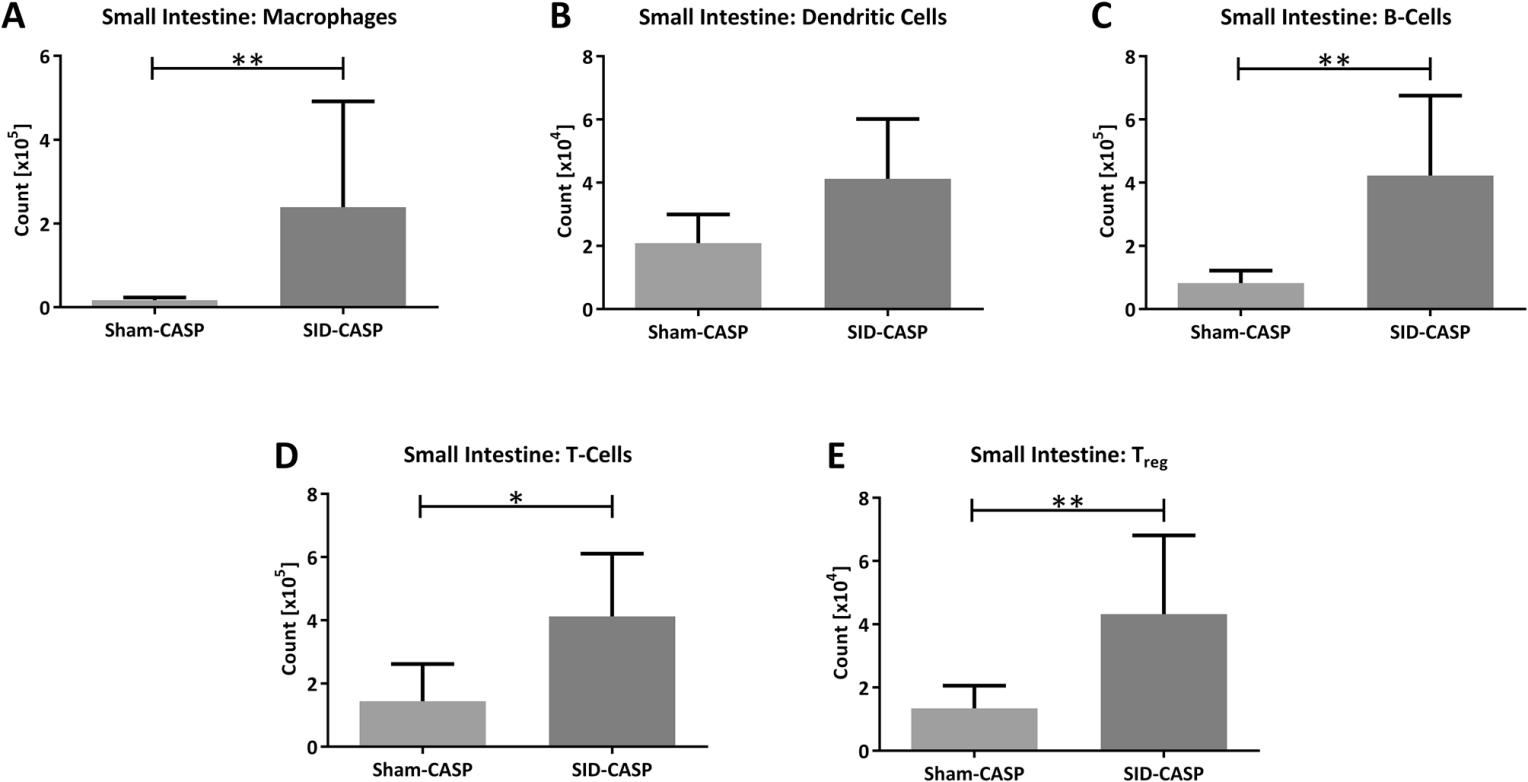
Infiltration of immune cells in small intestinal wall. C57BL/6 N mice underwent SID or a sham operation prior to 18G CASP. After 24 h the small intestine was physically dissolved and the cell suspension was stained for macrophages (**A**) (F4/80^+^), (**B**) dendritic cells (CD11c^+^), (**C**) B-cells (CD19^+^), (**D**) T-cells (CD3^+^) and (**E**) T_reg_-cells (CD3^+^,CD4^+^,FoxP3^+^) and analyzed using flow cytometry. N = 5 to 6(mice that have died before or during CASP procedure, as well as a technical failure have been excluded); Mann–Whitney U-Test; *p < 0.05; **p < 0.01.

We examined the small intestine for infiltration of immune cells using immunofluorescence and flow cytometry. Flow cytometry analysis showed significantly higher counts of macrophages, B-cells, T-cells, and T_reg_-cells in mice that underwent SID followed by CASP compared to sham followed by CASP (Fig. 5A, C–E). Dendritic cells showed no significant increase (Fig. 5B).

**Figure 6:**
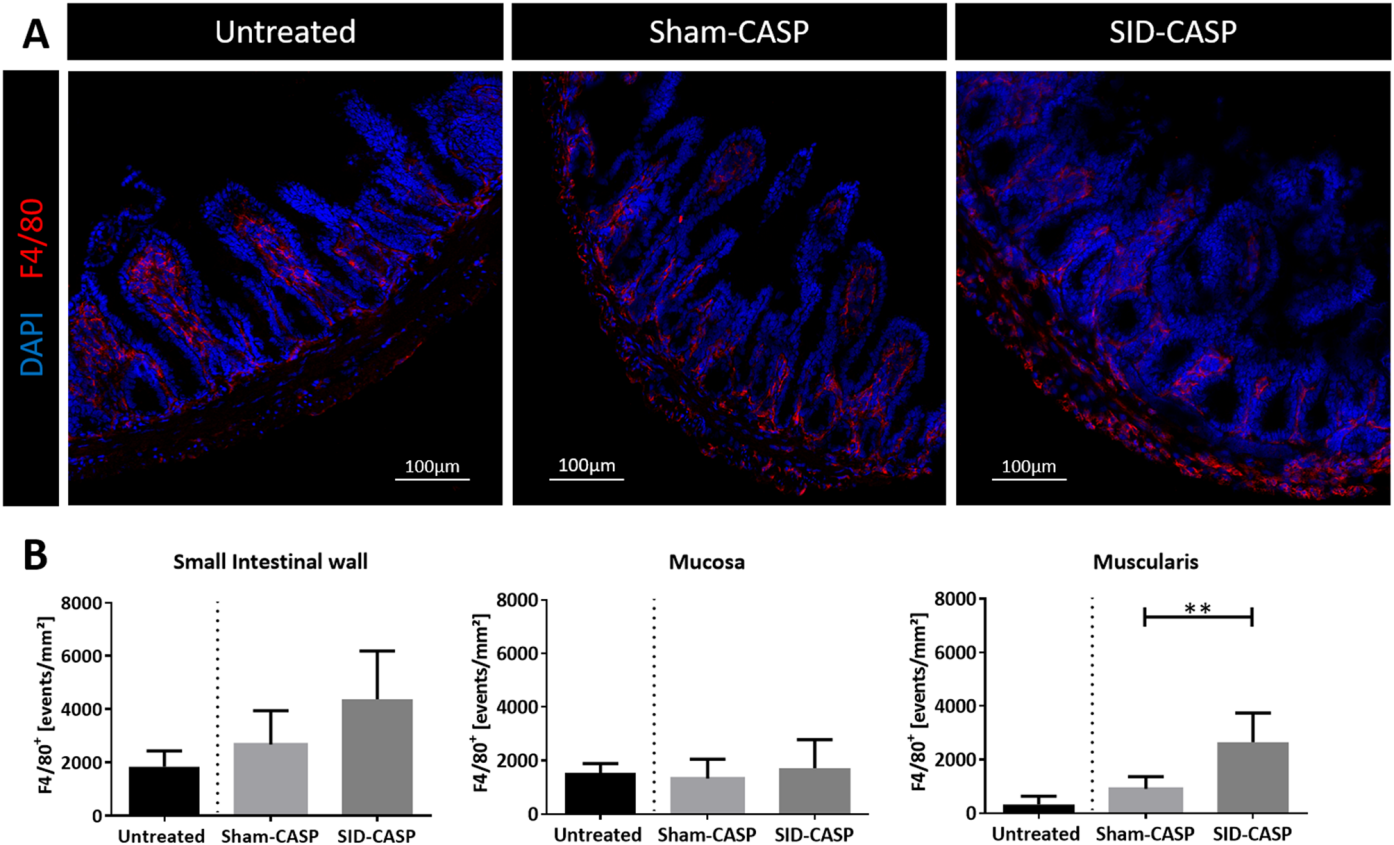
Representative immunofluorescence images of macrophages (F4/80^+^) in the small intestinal wall. C57BL/6 N mice underwent CASP following SID or a sham operation. (**A**) Aboral 1 cm of ileum was cryofixed and sections were stained for infiltrating macrophages using antibody against F4/80-antigen labelled with Alexa-647 dye and DAPI for staining of cell nuclei. Photos were taken from at least 6 mice per group. (Magnification 20x) (**B**) Fluorescence was assigned to the mucosal region and outer layers of muscularis and serosa (see materials and methods for detail). F4/80 positive events in (**B1**) whole cross section of small intestine, (**B2**) mucosa and (**B3**) muscularis were compared between operated groups. N = 6 to 8 (technical failures have been excluded); Mann–Whitney U-Test; **p < 0.01.

Immunofluorescence imaging for macrophage marker F4/80 confirmed cellular infiltration into the small intestinal wall after CASP. Each experimental group showed a specific distribution pattern of macrophages in the small intestinal wall. The images showed more positive signals of macrophages in the small intestinal wall of mice that underwent SID followed by CASP compared to animals that underwent previous sham as well as untreated control animals. This difference was most apparent in the muscularis region. Animals that underwent sham operation followed by CASP showed more positive signals compared to untreated mice. Positive signals in the sham group concentrated at the serosal edge and in the region of intestinal villi, corresponding to the mucosal region. In the muscular layer, only few signals could be detected in CASP mice with previous sham treatment. Untreated mice showed almost all F4/80 positive signals in the mucosal region (Fig. 6A).

To quantify these differences seen in immunofluorescence images between the groups, we manually separated the mucosal and muscular regions and quantified the F4/80 positive events present in each of these two sectors. We found no significant difference in the number of F4/80 positive events in the mucosal region between the groups. However, in the muscularis area, significantly higher counts of F4/80 positive events were found in animals that underwent SID followed by CASP, compared to previous sham (Fig. 6B).

## Discussion

Postoperative peritonitis is still a severe complication frequently seen in visceral surgery^1–3^. Among the sources of POP, late anastomotic leak is one of the most frequent^10,13^. This complications often translates into abdominal sepsis and is a major cause for postoperative deaths in visceral surgery^9^. During the last decades, the CASP model was widely used to investigate the changes in the immune system during abdominal sepsis. This model closely reflects the conditions seen in humans diffuse polymicrobial peritonitis^22^. The organism reacts with a strong pro-inflammatory response leading to a strong proinflammatory cytokine production with septic shock^22,30^. Simultaneously to this initial proinflammatory phase, a profound anti-inflammatory response with release of high systemic levels of IL 10 can be observed^22,39^. Additionally paraclinical parameters of multiple organ failure are increased^40^. Those findings reflect the immune conditions seen in humans suffering from severe abdominal sepsis^9,41^. However, CASP induces the leakage of stool synchronously to the surgical trauma^30^. In contrast, in surgical patients POP from anastomotic leak usually develops several days after initial surgical trauma^8^. To better model the immune conditions during the postoperative period at the onset of the anastomotic leak, the CASP model was combined with the SID model. This is the first report of this innovative combination of two well established models for different aspects of postoperative consequences on the immune system. The postoperative period is characterized by reduced HLA expression of human circulating monocytes, dysfunctional T-cells and impaired antibacterial responses^29,42,43^. Thus the murine SID model reflects the changes seen in human patients following surgery. SID mice also show low expression levels of MHC II on monocytes, lower lymphocyte counts with a relative increase of regulatory T cells and impaired ex vivo cytokine responses to LPS^12^. Additionally, SID induces an infiltration of immune cells into the gastrointestinal tract that might promote the development of postoperative ileus, a postoperative complication frequently seen in human visceral surgery^12,29^.

Our findings reported herein show that the combination of SID and CASP is a useful model to investigate the consequences of AL on the immune system.

Recent data indicate a protective role of the vagal nerve in murine sepsis models with increased survival through vagal stimulation and reduced survival in murine sepsis following vagotomy^31–34^. In those studies, the vagal nerve suppressed the proinflammatory cytokine response, and vagotomy led to increased systemic TNF α levels in mice that underwent LPS challenge or were submitted to abdominal sepsis models. However, in our model, vagotomy did not result in significantly increased mortality. In contrast to our experimental design, mice in other experiments underwent vagotomy and CASP simultaneously^31,32^. This may have increased the surgical trauma, resulting in a worsened outcome compared to our procedure, where mice had a recovery period between the two operations. Moreover, the strong impact of surgery preceding CASP induction (SID or sham) may have masked the influence of vagotomy in our model. Since subdiaphragmatic vagotomy did not alter the outcome of CASP in our experimental setting, we did not investigate its influence on the immune conditions in the following experiments.

In our model, SID induced a higher mortality of CASP compared to previous sham operation, as seen in patients suffering from POP compared to other forms of secondary peritonitis^10^. Regarding this, we analyzed the immune status of mice after SID alone. Seventy-two hours after SID, we observed reduced counts of peritoneal immune cells and found bacterial dissemination in various organs. This is consistent with observations in other intestinal manipulation models, which showed inflammation of the gastrointestinal tract following surgical manipulation, leading to alteration of mucosal permeability and bacterial overgrowth with consecutive translocation of bacteria after several days^44^. Bacteria and their compounds, such as LPS, can influence the local response to a subsequent infectious insult^45^. Depending on different factors, e.g., bacterial species, bacterial load, or the amount of LPS released during the first insult, hyper- or hyposensitivity to subsequent bacterial invasion can occur and alter cytokine responses and bacterial clearance^27,46,47^. Hypersensitivity might be beneficial in case of a second hit with few bacteria. In this case, the innate response might be more rapid and strong enough to clear the infection before harm to the infected organism occurs. In contrast, during a response against a high load of bacteria, hypersensitivity can lead to hyperinflammation and shock with a fatal outcome. Thus, Gumenscheimer et al. showed that mice sensitized with *Propionibacterium acnes* had increased survival after infection with a low dose of 10^5^ *Salmonella typhimurium*, but died rapidly when exposed to higher doses compared to mice without sensitization^48^. On the other hand, while hyposensitivity might prevent shock, the downregulation of the immune response can impair bacterial clearance and may cause an overwhelming infection^45,46^. However, in the case of secondary peritonitis, a controlled and sufficient immune response is essential, and alterations in any direction may worsen the outcome^41^.

In our experimental model, we found more pronounced inflammatory changes in the intestinal wall in animals that underwent SID followed by CASP than in mice with sham operation followed by CASP. Immunofluorescence additionally revealed different patterns of immune cell infiltration into the small intestinal wall. Previously SID-operated mice showed infiltrating monocytes in all layers of the small intestine. In contrast, previously sham-operated mice showed most infiltrating monocytes in the mucosal area and at the serosal side, but almost none in the muscularis area. It is described that during peritonitis, macrophage infiltration into the mucosa occurs^49^. In contrast, after intestinal manipulation, a macrophage network is activated inside the muscularis, and this layer becomes the main target of infiltrating cells^49,50^. We propose that the infiltration pattern found in previously sham-operated mice reflects cellular infiltration induced by CASP. In contrast, SID induced an infiltration that was already ongoing at the time of CASP induction and the resulting peritonitis modified the process initiated by SID. In consequence, an exacerbated inflammation further increased mucosal permeability and bacterial translocation. Also, the strong inflammation might have induced anti-inflammatory mechanisms to prevent tissue damage, thus explaining the higher counts of regulatory T cells in the small intestine in SID followed by CASP. In line with this, analysis of peritoneal lavage revealed higher levels of IL 10 after SID followed by CASP compared to sham operation followed by CASP, while comparable levels of IL 6, TNF α, and MCP-1 were found in both groups. During peritonitis, most IL 10 is produced by resident immune cells and cells recruited to the peritoneal cavity and gastrointestinal tract^41,51,52^. Also circulating monocytes show suppressive effects during abdominal sepsis by triggering the release of IL 10 from T cells^53^. We suppose those recruited and local immune cells to be the source of the higher IL 10 levels in our model.

There are various potential consequences of high IL 10 levels during abdominal sepsis. IL 10 might be crucial to prevent hyperinflammation in septic shock and reduces mortality during the hyperinflammatory phase of murine sepsis^54,55^. On the other hand, IL 10 impairs the antibacterial response and might cause death through overwhelming infection later in the course of the disease^27,52^. In seriously ill patients with general peritonitis and severe sepsis, induction of IL 10 occurs earlier and stronger than in less severe infections^41^. Therefore, the high levels of IL 10 found in the peritoneal lavage of SID pretreated CASP-animals could indicate a severe course of the disease with impaired bacterial clearance^39,56^. Lower immune cell counts in the peritoneal lavage suggest a further diminished antibacterial local response after SID followed by CASP, compared to previous sham-operated animals.

Based on our findings, we propose that bacterial translocation secondary to the initial surgical manipulation of the intestine and immune alterations directly induced by the initial operation are responsible for the increased severity of POP compared to other forms of secondary peritonitis. In our model, previous intestinal manipulation leads to severe inflammation of the small intestine, potentially resulting in bacterial spread and a first local immune response in the peritoneal cavity. This initial immune response translated into lower peritoneal immune cell counts and higher peritoneal IL 10, indicating an anti-inflammatory status after SID followed by CASP. This might prevent early deaths of CASP caused by hyperinflammation but could lead to overwhelming infection. This assumption is consistent with our survival analysis, showing that deaths mostly occurred later than 24 h after CASP.

## Conclusion

In summary, our data suggest subclinical bacteremia during the initial surgical procedure may induce an immunosuppressive local milieu in the peritoneal cavity, resulting in increased mortality after the occurrence of anastomotic leakage in POP. Moreover, the pattern of intestinal inflammation induced by peritonitis is modified by previous surgical manipulation, probably also contributing to the more severe clinical course. However, the "anti-inflammatory reflex" mediated by the vagal nerve had no impact on sepsis mortality in our model of POP, contrary to findings in other forms of sepsis.

Further research is warranted to investigate whether strategies directed against subclinical bacteremia during the initial surgical procedure, for example antibiotic prophylaxis or selective digestive decontamination, could be used to improve the outcome of POP.

## Methods

### Mice

Ten to fourteen-week-old female C57BL/6 N mice (weight ≥ 20 g) were bred by the Institute of Immunology and Transfusion Medicine, Greifswald, or purchased from Charles River (Wilmington, Massachusetts, USA). The mice were kept in groups of four to eight, ensuring that every cage contains at least one mouse per experimental group. After transfer in the animal operational tract within the building of Zentrale Service- und Forschungseinrichtung für Versuchstiere, University Medicine Greifswald, mice were allowed to acclimatize for at least 24 h. Mice had free access to water and food and cages were equipped with semi-naturalistic enrichment items. Daylight was set from 8 am to 8 pm with a constant temperature of 21 °C ± 2 °C. For analgesia, 2.5 mg Tramadol (Grünenthal GmbH, Stolberg, Germany) was added to 100 ml drinking water for the first postoperative week. Mice were anesthetized with ketamine (Selectavet Dr. Otto Fischer GmbH, Weyarn-Holzolling, Germany) (100 µg/g body weight) and xylazine (Selectavet Dr. Otto Fischer GmbH, Weyarn-Holzolling, Germany) (10 µg/g bodyweight) preoperatively or before euthanasia by cervical dislocation. Experimental group sizes were calculated based on a type 1 error of 0.05, a power of 0.8 and an effect size estimated using bibliographic data obtained in similar models. G*Power Version 3.1.9.2. (University of Kiel, Germany) was used for the calculation of the group sizes.

The ethics committee/institutional review board of the State Office for Agriculture, Food Safety and Fisheries, Mecklenburg-Western Pomerania (Landesamt für Landwirtschaft, Lebensmittelsicherheit und Fischerei Mecklenburg-Vorpommern, Veterinärdienste und Landwirtschaft; LALLF: 7221.3–1-046/15 and 7221.3–1.1–048/15) approved all procedures involving animals performed in this study. All experiments were performed under German animal safety regulations (Tierschutzgesetz-TierSchG). All experiments involving animals were carried out in compliance with ARRIVE guidelines^57^.

### Experimental designs

The following experimental designs were used.

**Figure 7:**
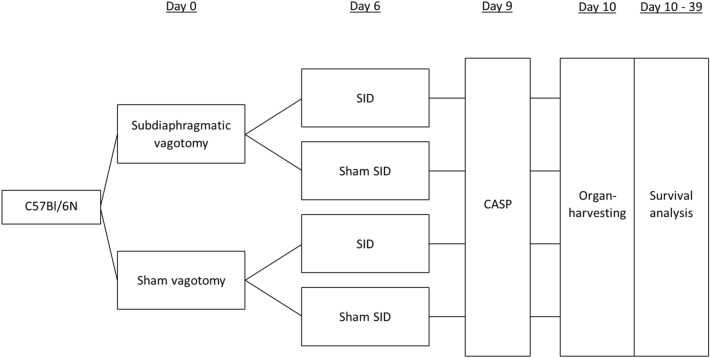
Experimental designs to determine the influence of vagotomy and SID on the course of peritonitis after CASP. C57BL/6 N mice underwent subdiaphragmatic vagotomy (VGX) or laparotomy (sham). After a recovery period of 6 days either *Surgically induced Immune Dysfunction (SID)* or another laparotomy (sham) was performed. 3 days later mice underwent 18G CASP. Survival of mice was controlled at least twice per day for the following 30 days or organs were harvested 24 h after CASP for analysis of immune parameters.

To determine if vagotomy or SID influence the survival after CASP, the experimental designs shown in Fig. 7 were used. Mice were randomly picked, marked at the tail to achieve an allocation to one of four groups. Afterward they underwent either subdiaphragmatic vagotomy or a sham vagotomy on day 0. Six days later, they were either subjected to SID or a sham operation. During SID or Sham SID, all animals with previous VGX were evaluated for gastric dilatation that indicated successful vagotomy. Mice without dilatation were excluded from further procedures. On day 9, all mice underwent CASP. Technical failures and death during operation or anesthesia lead to exclusion of the animals from analysis. Afterward, animals were observed over a period of 30 days (experimental setting 1: survival analysis), or organs were harvested 24 h after CASP (experimental setting 2) (Fig. 7).

**Figure 8:**
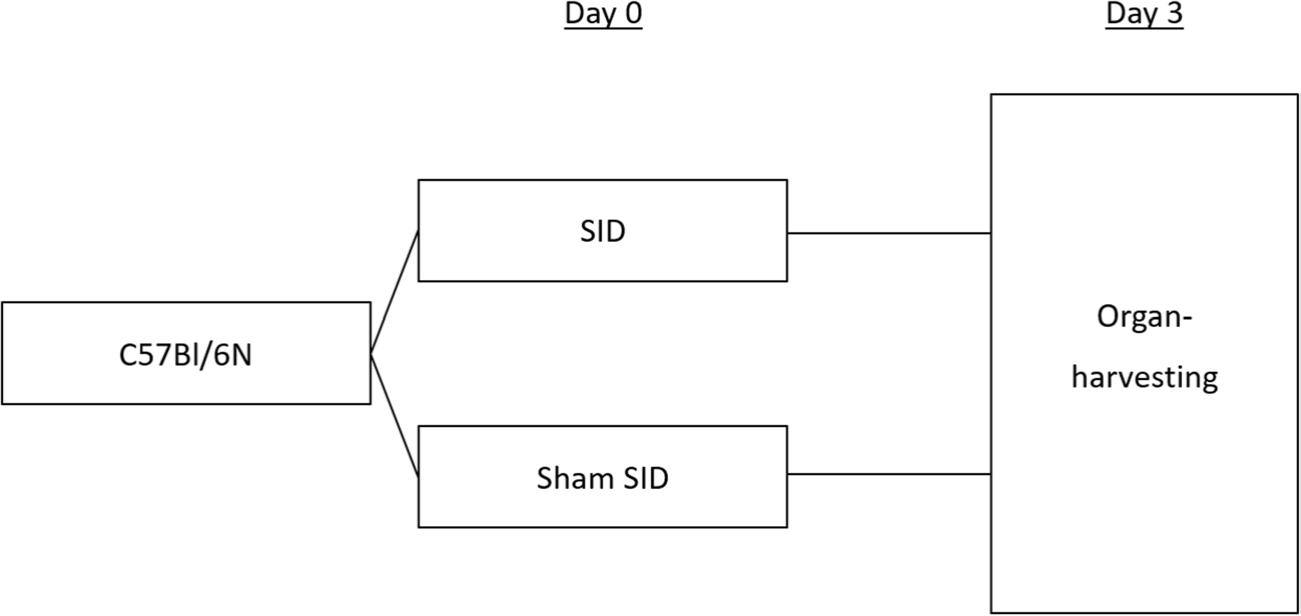
Experimental design to examine the alterations in the local immune response and bacterial spread due to SID. C57BL/6 N mice underwent either *Surgically induced Immune Dysfunction (SID)* or a laparotomy (sham). Three days later, organs were harvested for analysis of immune parameters.

The aim of the third experimental setting was the assessment of the peritoneal immune status directly before CASP (72 h after SID or Sham). Therefore, either SID or sham SID was performed and the local immune conditions in the peritoneal cavity and the bacterial spread were examined three days later (Fig. 8).

### Subdiaphragmatic vagotomy

Subdiaphragmatic vagotomy was performed as a modified technique described by Kessler et al*.*^32^. Mice were anesthetized with ketamine (Selectavet Dr. Otto Fischer GmbH, Weyarn-Holzolling, Germany) (100 µg/g body weight) and xylazine (Selectavet Dr. Otto Fischer GmbH, Weyarn-Holzolling, Germany) (10 µg/g bodyweight). The upper abdominal wall was opened through a midline incision. The liver and abdominal wall were kept out of the operation field with small tissue hooks (Braintree Scientific Inc., Braintree, USA). All subsequent steps were performed under a surgical microscope (Leica Camera AG, Wetzlar, Germany). The esophagus was mobilized on its hepatic side and lifted. Then, the ventral and dorsal branches of the vagal nerve were exposed and transected. After intraabdominal injection of 0.5 ml of sterile saline, the abdominal wall was closed with continuous sutures, using 5/0 Vicryl (Johnson&Johnson, New Brunswick, USA) for the fascia and 4/0 Polyester (Catgut, Markneukirchen, Germany) for the skin. Successful vagotomy was verified by the presence of gastric dilatation when relaparotomy was performed six days later. Only mice with a gastric dilatation were used in further experiments. Control mice underwent laparotomy, and the abdomen was closed after the average time of the vagotomy procedure.

### Surgically induced immune dysfunction (SID)

As previously described^12^, mice were anesthetized (ketamine (100 µg/g body weight) and xylazine (10 µg/g body weight)), and a midline laparotomy was performed. The small intestine was identified and gently squeezed between two cotton swabs three times to imitate an intraoperative manipulation of the gut. Afterward, 0.5 ml of sterile saline was added to the peritoneal cavity, and two continuous sutures were performed as described above. The sham group underwent the same procedure without mobilization and manipulation of the small intestine.

### Colon ascendens stent peritonitis (CASP)

CASP was performed as previously described^30^. After disinfection of the abdominal skin, a midline laparotomy was performed. The ascending colon was exposed, and an 18G cannula (BD Venflon, Becton Dickinson, Franklin Lakes, NJ, USA) was inserted into the antimesenteric wall of the ascending colon. The cannula was fixed using single stitches with 7/0 Meriderm (Catgut, Markneukirchen, Germany). Then stool was pressed from the ascending colon through the stent. Afterward, 0.5 ml of sterile saline was injected into the peritoneal cavity before the closure of the abdominal walls with two continuous sutures as described above. For additional analgesia, mice were injected with 0.5 ml saline with 2 µg buprenorphine s.c. at the end of CASP procedure. After CASP, mice were controlled every six to eight hours until the second postoperative day and once per day afterward. Clinical status was defined with a standardized scoring system (Supplemental Table 2). Mice with signs of critical disease (Score ≥ 9) were euthanasized.

### Bacteriological analysis of various organs

Blood was harvested by retroorbital puncture 72 h after SID or sham (day 0). After death by cervical dislocation, the mice were externally disinfected by immersion into 70% alcohol. Peritoneal lavage was performed with 10 ml sterile PBS. Liver, spleen, left kidney, and lung were collected and homogenized using a Precellys 24 Homogenizer (Bertin Technologies SAS, Montigny-le-Bretonneux, France). Organ homogenates, blood and peritoneal lavage were diluted (1, 1:10, 1:100 and 1:1000) and applied onto blood agar plates. Those were incubated at 37 °C for 22 h, and colonies were counted. Colony-forming units (CFU) per gram of organ weight or milliliter blood and peritoneal lavage were determined.

### Flow cytometry of peritoneal lavage and small intestine

The peritoneal lavage was centrifuged and sediments were washed with PBS (Thermo Fisher Scientific Inc., Waltham, MA, USA). The whole small intestine was explanted and rinsed several times with pre-cooled PBS. Then, small pieces were cut and homogenized using a Precellys 24 Homogenizer (Bertin Technologies SAS, Montigny-le-Bretonneux, France). The suspension was filtered through a 70-µm cell Easystrainer (Greiner Bio-One, Kremsmünster, Austria), and gradient centrifugation was performed with 40% and 70% Percoll (Thermo Fisher Scientific Inc., Waltham, MA, USA). Interphases were collected and washed with PBS.

Cell numbers were determined using BD TruCount Beads (Becton Dickinson, Franklin Lakes, NJ, USA) according to the manufacturer's protocol. 2 × 10^6^ cells were used for the following analysis. Fc-blockage was performed using Fc-Block (Miltenyi Biotec, Bergisch Gladbach, Germany). Antibodies against B220, CD4, CD11c (all from Becton Dickinson), CD19, CD11b, MHC-II, and Ly6G (all from Thermo Fisher Scientific Inc), F4/80, FoxP3, CD25, CD4, CD69, and CD8 (all from BioLegend, San Diego, CA, USA), as well as a secondary antibody against streptavidin (Thermo Fisher Scientific Inc) were used for the analysis. For FoxP3-staining, permeabilization was performed using the True-Nuclear Transcription Factor Buffer Set (BioLegend) according to the manufacturer's protocol. Samples were analyzed using a BD LSR-II cytometer (Becton Dickinson) and data were analyzed using FlowJo software (Becton Dickinson). T-helper cells were defined as CD3^+^ and CD4^+^, cytotoxic T-cells as CD3^+^ and CD8^+^, and regulatory T-cells (T_reg_) as CD3^+^, CD4^+^ and FoxP3^+^. B-cells were defined as B220^+^ and CD19^+^, neutrophile granulocytes as Ly6G^+^, dendritic cells as CD11c^high^ and MHC-II^+^ and peritoneal macrophages as F4/80^+^ or CD11b^+^ after gating of all previously mentioned cell types with their specific antigens. Intestinal macrophages and inflammatory monocytes in small intestinal tissue were defined as F4/80^+^.

### Immunohistochemical analysis of the small intestine

The whole small intestine was explanted, and 1 cm of the aboral end was cut and frozen in kryoTec freezing medium (Kryotec-Kryosafe GmbH, Hamburg, Germany). Five-µm slices were cut and fixed with acetone. Staining of inflammatory monocytes was performed with F4/80 antibody (AbD Serotec, Kidlington, UK) and secondary antibody goat anti-rat A647 (abcam plc. Cambridge, UK). DAPI (Thermo Fisher Scientific Inc) was used to counterstain cell nuclei. For histological analysis, images were recorded as 24-bit TIFF files on a Keyence BZ-9000® fluorescence microscope (Keyence Corporation, Osaka, Japan) with 20 × magnification, then saved using the BZ-II Image Viewer (Ver. 1.41, Keyence Corporation) and Analyzer (Ver. 1.42, Keyence Corporation). Cell segmentation was determined using a "positive cell detection" algorithm in QuPath (0.2.0-m4, University of Edinburgh, Scotland) with cell categorization by cytoplasm^58^. Data were compiled using jupyterlab (1.2.5) with the following packages: Python (Ver. 3.6.8), Numpy (Ver. 1.18.1) and Pandas (Ver. 0.24.0). Each biological replicate was calculated by the mean of three technical replicates.

### Cytokine measurement

The peritoneal lavage was centrifuged for 5 min at 300 × g and 4 °C. The supernatant was harvested and kept in aliquots at − 80 °C until cytokines were measured using a custom panel CBA (Becton Dickinson, Franklin Lakes, USA) with IFNy, IL 1ß, IL 4, IL 6, IL 10, TNF α, MCP-1 and IL 17 according to the manufacturer's protocol. Calculation of cytokine levels was performed with the FCAP Array Software (Becton Dickinson, Franklin Lakes, USA) according to manufacture’s protocol.

### Statistical analysis

Statistical analyses were performed in GraphPad Prism6 Software (GraphPad Software, San Diego, USA). Differences in survival rates were assessed using the log-rank test. Statistical outliers were identified by ROUT method with Q set to 1% and excluded from analysis. Comparisons from cytokine measurements, immunofluorescence and flow cytometry were done using the two-tailed Mann–Whitney U-test for nonparametric probes or the Kruskal–Wallis test combined with Dunn's test. P-levels equal to or below 0.05 were considered statistically significant.

## Supplementary Information

Supplementary Information.
