# Supplementary material for: Increased mortality and altered local immune response in secondary peritonitis after previous visceral operations in mice
Source: Sci Rep. 2021 Aug 10;11:16175. doi: 10.1038/s41598-021-95592-5 (PMC8355121; doi:10.1038/s41598-021-95592-5)
Supplement: Supplementary file 1 — Supplementary Information. [file 41598_2021_95592_MOESM1_ESM.docx]

**Supplemental Information:**

**Supplemental Table 1: Multiple comparison of survival after CASP**

| Comparison | Log-rank p-value | difference of survival |
| --- | --- | --- |
| Sham-Sham-CASP x VGX-Sham-CASP | 0.6 | ns |
| Sham-SID-CASP x VGX-SID-CASP | 0.8 | ns |
| Sham-Sham-CASP x Sham-SID-CASP | 0.02 | * |
| Sham-Sham-CASP x VGX-SID-CASP | 0.009 | ** |
| VGX-Sham-CASP x Sham-SID-CASP | 0.002 | ** |
| VGX-Sham-CASP x VGX-SID-CASP | 0.002 | ** |

^Log-rank test; *p<0.05; **p<0.01^

Multiple comparison of survival revealed significant changes due to SID procedure. Every experimental group that underwent SID showed significantly lower survival rates compared with any group that underwent Sham. Comparison between the two groups with Sham as well as the two groups with SID procedure showed no significant differences.

**Supplemental Table 2**

| **Parameters** | **Assessment** | **Criteria** | **Points** |
| --- | --- | --- | --- |
| External appearance | Observation | - smooth, well-groomed fur  - ruffled fur  - damp four  - slimy eyes | 0  1  2  3 |
| Respiration | Observation | - normal  - moderate tachypnoea  - severe tachypnoea  - week respiratory excursions, ataxic respiration | 0  1  2  3 |
| Weigth loss | Balance | <5%  <15%  <20 %  >20% | 0  1  2  3 |
| Spontaneous behaviour | Observation | - normal, lively, curious  - reduced spontaneous activity, hunched back  - inactive, unsteady gear  - lateral position | 0  1  2  3 |
| Provoked behaviour | Observation | - escape reaction when cage is opened  - escape reaction when hand is approaching  - escape reaction on manual contact  - no escape reaction | 0  1  2  3 |
| Abdominal palpation | Palpation | - soft, no pain reaction  - tender, minimal pain reaction  - resistance, frank pain reaction  - tense abdomen | 0  1  2  3 |
| Appearance of feces | Inspection | - sufficient quantity of formed stool  - sufficient quantity of unformed stool  - reduced quantity of stool  - no fresh stool | 0  1  2  3 |

**Scoring system SID /CASP**
